# Supplementary material for: A Critical View of the Application of the APEX Software (Aqueous Photochemistry of Environmentally-Occurring Xenobiotics) to Predict Photoreaction Kinetics in Surface Freshwaters
Source: Molecules. 2019 Dec 18;25(1):9. doi: 10.3390/molecules25010009 (PMC7017383; doi:10.3390/molecules25010009)
Supplement: Supplementary file 1 [file molecules-25-00009-s001.zip › 3.2.4_gcc-4.4.0.zip!/3.2.4_gcc-4.4.0/3.2.4_gcc-4.4.0/doc/octave/html/interpreter/Operator-Precedence.html]

Operator Precedence - Untitled


Previous: Increment Ops,
Up: Expressions


---

### 8.8 Operator Precedence

Operator precedence determines how operators are grouped, when
different operators appear close by in one expression. For example,
\* has higher precedence than +. Thus, the expression
`a + b * c` means to multiply `b` and `c`, and then add
`a` to the product (i.e., `a + (b * c)`).

You can overrule the precedence of the operators by using parentheses.
You can think of the precedence rules as saying where the parentheses
are assumed if you do not write parentheses yourself. In fact, it is
wise to use parentheses whenever you have an unusual combination of
operators, because other people who read the program may not remember
what the precedence is in this case. You might forget as well, and then
you too could make a mistake. Explicit parentheses will help prevent
any such mistake.

When operators of equal precedence are used together, the leftmost
operator groups first, except for the assignment and exponentiation
operators, which group in the opposite order. Thus, the expression
`a - b + c` groups as `(a - b) + c`, but the expression
`a = b = c` groups as `a = (b = c)`.

The precedence of prefix unary operators is important when another
operator follows the operand. For example, `-x^2` means
`-(x^2)`, because - has lower precedence than ^.

Here is a table of the operators in Octave, in order of increasing
precedence.

`statement separators`: ;, ,. `assignment`: =, +=, -=, \*=,/=. This operator groups right to left. `logical "or" and "and"`: ||, &&. `element-wise "or" and "and"`: |, &. `relational`: <, <=, ==, >=, >, !=, ~=. `colon`: :. `add, subtract`: +, -. `multiply, divide`: \*, /, \, .\, .\*, ./. `transpose`: ', .' ``` unary plus, minus, increment, decrement, and ``not'' ```: +, -, ++, --, !, ~. `exponentiation`: ^, \*\*, .^, .\*\*.
